# Structural and Computational Characterization of Disease-Related Mutations Involved in Protein-Protein Interfaces

**DOI:** 10.3390/ijms20071583

**Published:** 2019-03-29

**Authors:** Dàmaris Navío, Mireia Rosell, Josu Aguirre, Xavier de la Cruz, Juan Fernández-Recio

**Affiliations:** 1 Barcelona Supercomputing Center (BSC), 08034 Barcelona, Spain; damarisnavio@gmail.com (D.N.); mireia.rosell@bsc.es (M.R.); 2 Vall d’Hebron Institute of Research (VHIR), Universitat Autònoma de Barcelona, 08035 Barcelona, Spain; josu.aguirre@vhir.org (J.A.); xavier.delacruz@vhir.org (X.d.l.C.); 3 Institució Catalana de Recerca i Estudis Avançats (ICREA), 08010 Barcelona, Spain; 4 Institut de Biologia Molecular de Barcelona (IBMB), Consejo Superior de Investigaciones Científicas (CSIC), 08028 Barcelona, Spain; 5 Instituto de Ciencias de la Vid y del Vino (ICVV), CSIC—Universidad de La Rioja—Gobierno de La Rioja, 26007 Logroño, Spain

**Keywords:** protein-protein interactions, single amino acid variants, structural bioinformatics, computational docking, interface prediction

## Abstract

One of the known potential effects of disease-causing amino acid substitutions in proteins is to modulate protein-protein interactions (PPIs). To interpret such variants at the molecular level and to obtain useful information for prediction purposes, it is important to determine whether they are located at protein-protein interfaces, which are composed of two main regions, core and rim, with different evolutionary conservation and physicochemical properties. Here we have performed a structural, energetics and computational analysis of interactions between proteins hosting mutations related to diseases detected in newborn screening. Interface residues were classified as core or rim, showing that the core residues contribute the most to the binding free energy of the PPI. Disease-causing variants are more likely to occur at the interface core region rather than at the interface rim (*p* < 0.0001). In contrast, neutral variants are more often found at the interface rim or at the non-interacting surface rather than at the interface core region. We also found that arginine, tryptophan, and tyrosine are over-represented among mutated residues leading to disease. These results can enhance our understanding of disease at molecular level and thus contribute towards personalized medicine by helping clinicians to provide adequate diagnosis and treatments.

## 1. Introduction

Large-scale sequencing initiatives such as the 1000 Genomes Project [1] and the Exome Sequencing Project together with the important drop experienced by next-generation sequencing (NGS) costs [2] have provided a significant source of genomic information for an increasing number of healthy individuals and patient populations. In this context, understanding the molecular-level impact of genetic variants and its relationship to disease would further contribute to bridging the gap between genotypes and phenotypes and thus improve methods of prevention, diagnosis, and treatment of pathological conditions [3].

The predominant source of genetic variations detected in human population comes from single nucleotide variants (SNVs), which imply one single base substitution in the genome. Among SNVs, those that result in amino acid substitutions at the protein sequence level might be associated with genetic diseases since they can alter protein stability, interfere with protein-protein interaction properties [4,5,6], eliminate catalytic activity, affect protein folding [7], or lead to aggregation [8]. It has been estimated that around 58% of the ~13,000 exonic SNVs carried per person lead to single amino acid variants (SAVs) [9].

Therefore, studying the effects of protein sequence variants on molecular function is crucial, but experimental methods are costly, time-consuming and challenging, making it infeasible to analyze a large number of amino acid substitutions. Hence, computational tools that rely on conservation-related attributes reflecting structural and functional relevance, as well as on protein structure and stability-related properties following the relationship between structure and function, are used to estimate the phenotypic effect of these variants. Some examples of such reported methods are SIFT [10], CADD [11], PolyPhen-2 [12], PON-P2 [13] or PMut [14]. However, pathogenicity predictors do not accomplish the requirements of clinical applications for standalone tools since they show success rates of only around 80% on average, with prediction rates dramatically lower for specific diseases [15,16]. It is evident that current predictors are not able to capture all the possible effects of mutations at the molecular level. For this reason, a more detailed description of these variants, including information such as their potential involvement in protein-protein interactions (PPIs), would help to improve the prediction of their pathogenic character, providing a more accurate representation of the association between genetic variants and their phenotype by complementing general predictive methods.

Recent studies show that mutations in protein-protein interfaces are over-represented among disease-causing mutations [17,18,19]. While common variants from healthy individuals rarely affect interactions, almost two-thirds of disease-associated mutations perturb PPIs. Half of these pathogenic variants are ‘edgetic’ mutations, which impair only a subset of interactions while leaving most others unperturbed [20]. Consequently, within the context of PPI networks, knowledge about the molecular mechanisms by which genetic variants affect interaction networks can elucidate how mutations on the same gene might cause different phenotypes [6].

Regarding disease-causing mutations at PPI interfaces, they can induce geometrical and physicochemical changes at interaction sites that may affect interface stability, interface conformation dynamics through disruption or stabilization of specific conformational states, and the direct interactions between partner protomers [19]. Thus, the structural location of PPI interface mutations is important concerning pathogenicity. It has been demonstrated that disease-causing protein sequence variants are preferentially located in the solvent-inaccessible interface zones (‘core’), as opposed to the interface regions that remain partially solvent accessible (‘rim’) and are enriched in polymorphisms in the same way as the non-interacting surface. Moreover, energetic hot-spot residues, which play a crucial role in the free binding energy of the complex, tend to be enriched in disease-causing mutations regardless of the interface location [18]. All these findings highlight the importance of understanding the effects of protein sequence variants in protein structure to grasp the genotype to phenotype relationships.

In this study, we have characterized protein-protein interactions involving 58 proteins with pathogenic mutations related to diseases detected in newborn screening. Interpretation of mutational data in this set of proteins is of major clinical interest regarding the possibility of large-scale gene sequencing to detect disorders in newborn testing. We used the experimentally solved structures of the protein complexes when available, and when not, the protein-protein interface was predicted by an ab-initio docking approach. The distribution of disease-causing and neutral variants across the different interface regions, as well as the substitution susceptibility of distinct amino acids, was discussed.

## 2. Results

### 2.1. Structural Characterization of Proteins and Interactions in Diseases Detected in Newborn Screening

A total of 58 proteins with pathogenic mutations involved in diseases detected in newborn screening were analyzed (Table S1). As many as 56 of these proteins had structural information in Interactome3D, from which 42 had more than 80% structural coverage (Table S2). Only 16 of these proteins were monomers; 35 were homo-oligomers, and 5 hetero-oligomers. There were experimental structures for 62% of these 42 proteins, while for the remaining 38% of proteins, the Interactome3D database provided a homology-based model.

Among the 58 analyzed proteins, 50 of them had interaction data available in Interactome3D database (although three of them form only self-interactions). From the total of 389 PPIs found in such databases, only a small fraction (<12%) had available complex 3D structure. All these PPIs involved a total of 351 protein partners, the majority of which (75%) had available 3D structure. Among the partners with known 3D structure, 37% of them had good structural coverage (>80% of its sequence) in a single PDB file, while 40% of them have their structure split in separated PDB files.

**Figure 1:**
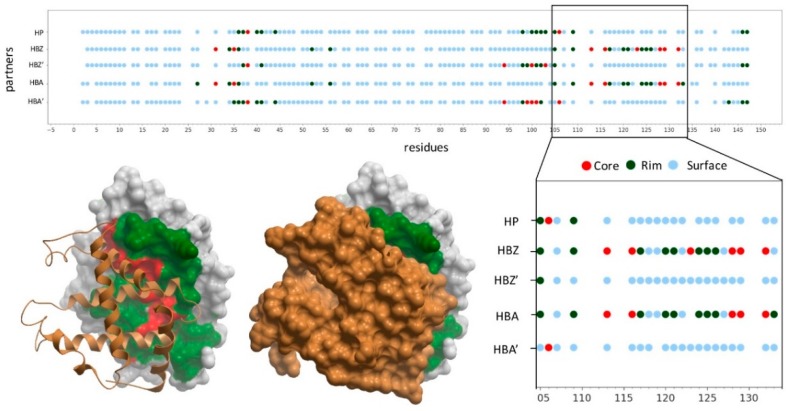
Structural characterization of hemoglobin subunit beta (HBB) interactions. The graphic represents the binding interface of HBB with different partners for which there is an available complex structure: haptoglobin (HP, hemoglobin subunit zeta (HBZ) and hemoglobin subunit alpha (HBA). As HBB interacts with both HBA and HBZ forming a heterotetramer, two different interfaces are formed with each of the HBB subunit (HBB-HBA and HBB-HBA’, or HBB-HBZ and HBB-HBZ’). The graphic represents as dots the non-interacting surface residues (in blue), the interface rim residues (in dark-green) and the interface core ones (in red). The complex structure between HBB (white skin) and HBZ (gold ribbon or skin) is represented, with HBB interface rim residues (in green) and interface core ones (in red).

Protein-protein interfaces were divided into core and rim residues (see Methods). For a given protein, residues can be defined as surface, interface core or interface rim depending on the considered partner. Figure 1 shows an example of a protein in which there is an available structure for the majority of its interactions, and the residues have been annotated according to such interaction data. Interface patches in one protein can be the same for some partners and different for others. This is important, since protein sequence variants in these regions could disrupt only a subset of interactions, possibly leading to ‘edgetic’ effects [20].

In all the annotated PPIs involving the 58 analyzed proteins, there were a total of 11,199 residues, of which 6019 were found to be buried in at least one structure (Table 1). Of the remaining non-buried (surface) residues, 2062 were located at the interface with at least one protein partner, and of these, 1146 residues were found at the interface core at least in one complex.

### 2.2. Residues Energetically Relevant for the Interaction Are More Likely to Be at the Interface Core

The energetic contribution to protein complex stability was not uniform across the interface, and for instance, interface residues that were estimated to be energetically relevant for the interaction (i.e., those with residue binding energy < -2 a.u., as calculated by pyDock) tended to be located at the interface core region more often than expected by random (Table 2). This was consistent with previous studies reporting that core amino acids contribute significantly more than rim amino acids to the binding free energy of the complex [18,21].

### 2.3. Pathogenic and Neutral Variants Are Differentially Distributed in Protein-Protein Interfaces

A total of 2882 disease-causing mutations and 1552 neutral variants were mapped onto the 3D structures of the protein-protein interactions involving the 58 genes analyzed here (Table 1). Around 47% of all variants occurred in solvent-accessible residues, which included non-interacting regions (surface) and interacting ones (interface).

Regarding the disease-causing variants, 36% of them (1040) occurred in solvent-accessible residues, of which 488 were found at the interface with at least one partner (with 337 of them at the core region in at least one complex). The odds of being located at the interface rather than at a non-interacting surface was 1.44 higher for pathogenic variants compared to the rest of residues (OR 1.44, 95% CI 1.25–1.54, *p* < 0.0001), which is consistent with previous studies [4,17,18,21]. More specifically, the odds of being located at the interface core region rather than rim was 2.11 higher for disease-causing variants compared to the rest of the residues, similar to the odds of being located at the interface core rather than at non-interacting protein surface (1.94). On the other side, there was no significant difference between the location of disease-causing variants at the interface rim region and the non-interacting protein surface (Table 1). These results show clearly the different impact of interface core and rim mutations in human disease.

Regarding the neutral variants, 66% of them occurred in solvent-accessible residues, of which 261 were found at the interface region (being 83 of them at the core, and 178 at the rim), and 767 at the non-interacting protein surface. Contrarily to disease-causing variants, the odds of being located at the interface rather than at a non-interacting protein surface was smaller for neutral variants compared to the rest of residues (OR 0.44, 95% CI 0.38–0.52, *p* < 0.00001). Moreover, for these neutral variants, the odds of being located at the interface core rather than the rim or the non-interacting surface was 0.32 and 0.24, respectively. As in the case of disease-causing variants, there was no significant difference between the location of neutral variants at the interface rim region and the non-interface protein surface.

**Figure 2:**
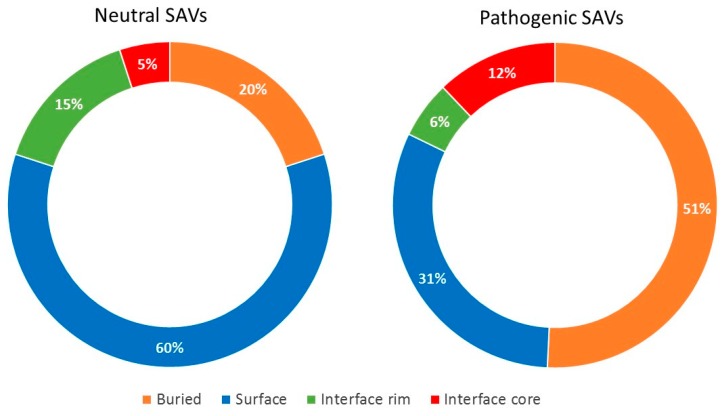
Structural characterization of residues in HBB affected by neutral or pathogenic variants. The graphics show the percentage of HBB residues affected by either neutral or pathogenic variants, as a function of their location in the available protein-protein complex structures.

The division of the interface into core and rim regions showed that the core was enriched in disease-causing variants, while the rim was enriched in neutral variants. As in the case of the non-interacting protein surface, amino acid changes in the rim region can be easily accommodated without significant distortions in the overall fold of the protein and without affecting the PPIs. This was consistent with their lower evolutionary conservation and higher side-chain flexibility [22]. Figure 2 shows the distribution of neutral and pathogenic SAVs in a case example (HBB, in which there is available structure for the majority of its interactions). There were five PPIs annotated in Interactome3D for HHB. Four of these PPIs had an available structure (or reliable model): HBB-HP, HBB-HBA1, HBB-HBZ and HBB-HBB. As can be seen, the proportion of pathogenic variants located in the interface rim and the non-interacting surface was much lower than the proportion of neutral variants in the same regions.

### 2.4. Amino Acid Substitution Susceptibility in the Interface Is Larger in Pathogenic Variants

**Figure 3:**
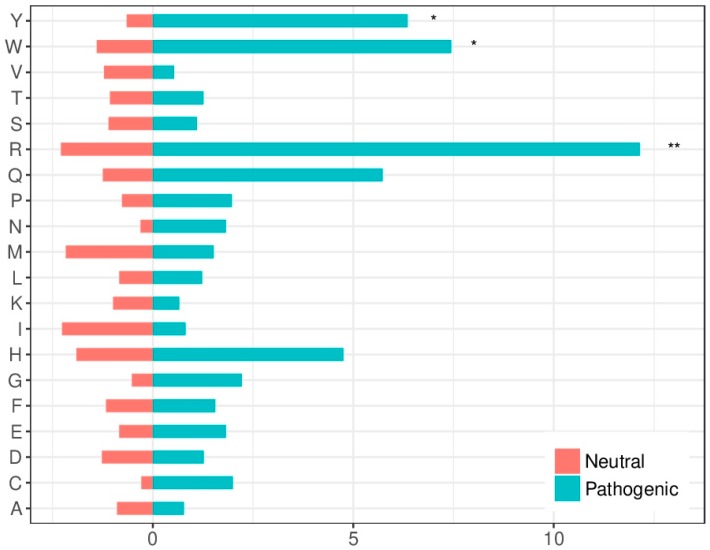
Amino acid substitution susceptibility to disease-causing or neutral variants within protein interfaces. The bars show the percentage of residues of a given type that are found mutated in disease-causing variants (in blue) or neutral variants (in red). Asterisks show statistical significance according to a “*N-1*” χ^2^ test (* 0.01 < *p* < 0.05; ** 0.001 < *p* < 0.01).

The amino acids mutability susceptibility was analyzed to determine whether it could be relevant for the molecular characterization of disease-causing variants. Arginine (R) was the most mutated residue in both neutral and pathogenic variants in protein-protein interfaces. This high mutability can be explained by the fact that four out of the six codons for R include CpG dinucleotides, which tend to mutate at rates 10–15 times higher than other dinucleotides in the DNA [23]. Arginine (R), tryptophan (W) and tyrosine (Y) were significantly over-represented among mutated residues leading to disease (Figure 3), which is coherent with previous findings [24].

### 2.5. Docking-Based Interface Prediction for Further Characterization of Protein Sequence Variants: A Case Study

Many of the proteins analyzed here are involved in protein-protein interactions for which there is no available complex structure. In these cases, we could apply docking simulations to identify potential interface residues, using a pyDock approach and NIP interface prediction module. First of all, in order to apply docking simulations, we needed to check whether we had a complete structure or a reasonable model for the interacting proteins. Available databases such as Interactome3D provide this information. However, there are several issues to consider here. For example, in many cases either a model or an experimental version of the overall structure of the target protein is available, but with incomplete structural coverage. Or, it may also happen that the overall structure is split between different PDB files, and then we would need to infer the global structure from these different parts, a non-trivial problem. In Table S2 we identified those proteins that have >80% structural coverage in a single PDB file. This global structure would be suitable for docking. If this global structure is not available, because of incomplete coverage or because it is split between different PDB files, we could still use for docking the individual domains that have >80% structural coverage, and then we would try to rebuild the whole protein in the context of the docking models. Since this is out of the scope of automatic docking, we have not used these cases here for docking. In addition to the previous issues, it is important to identify the oligomeric state of the interacting proteins, so that we use for docking the correct assembly form. Table S2 provides such information.

**Figure 4:**
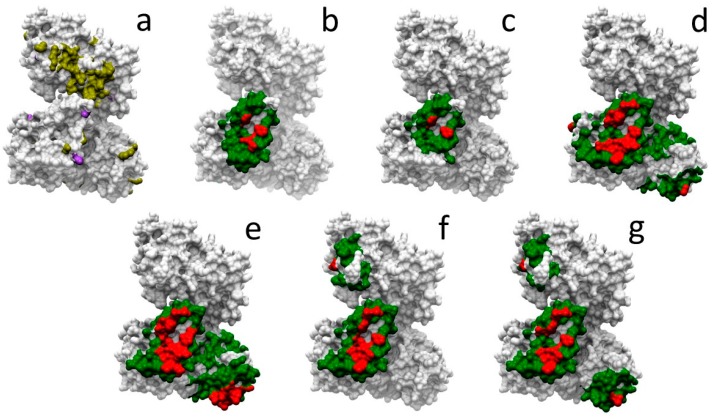
Docking-based characterization of HADHA mutations related to protein interactions. (**a**) Neutral (yellow) and disease-related (purple) mutations mapped on HADHA structure. In panels (**b**–**g**), docking-based predicted interface core (red) and rim (green) residues in HADHA for the interaction with the following partners: (**b**) O95166, (**c**) P60520, (**d**) Q14164, (**e**) Q99714, (**f**) Q9GZQ8, and (**g**) Q9H0R8.

Given all the above considerations, we have selected one example case, HADHA, involved in 13 different protein-protein interactions for which there is no complex structure available. There are 116 neutral and 31 pathogenic mutations described in HADHA. Figure 4a shows the location of these mutations in HADHA structure. However, they cannot be located at any protein-protein interface due to the lack of this structural information. We explored whether docking-based estimation of interface residues could help to further characterize such mutations. In six of these interactions, interacting partners have sufficient structural coverage (i.e., >80%) for docking. We used pyDock to run docking in these cases, and based on that, we estimated the interface rim and core residues from the NIP calculations. Figure 4b–g shows the predicted interface core and rim residues for each of these interactions, which can be used to visually check whether any of the known variants in HADHA are located at the predicted interfaces. Table 3 shows in detail the neutral and pathogenic SAVs in HADHA that are located at the different predicted interfaces. Disease-related mutations R399*, Y740* and V412L, involved in mitochondrial trifunctional protein deficiency, are found in all predicted interfaces. Pathological mutations with more specific effects are Q358K, involved in haemolysis, elevated liver enzymes, and low platelets, and found at the predicted interface with Q14134 (Figure 4d), or R610K, involved in long-chain 3-hydroxyacyl-CoA dehydrogenase deficiency, and found at the predicted interface with Q14134 and Q99714 (Figure 4d,e).

## 3. Discussion

To better understand the functional influence of genetic variants at the protein level, structural characterization of single amino acid variants and their interactions is one of the basic steps. In this regard, several structural databases of protein interaction data can be used, such as Interactome3D [25]. However, a major limitation is the low availability of 3D structures for protein-protein complexes. Consequently, a large fraction of protein sequence variants cannot be precisely located at protein interfaces. For this reason, using docking models to estimate whether variants can be involved in PPIs may be auspicious. In this regard, a potential problem for the application of docking at a large-scale is that most of the available interaction databases essentially provide sets of binary interactions (i.e., protein-protein interacting pairs), while for this type of experiment we would need more detailed data, such as the identification of the contacting domains, or the oligomeric state of the interacting proteins. We have collected all this information for the proteins and interactions analyzed here (Table S2). This can be valuable information in order to run docking simulations in the most realistic conditions. To test this in a real example, we chose HADHA interactions, in which interacting partners had available 3D structure with >80% structural coverage. We applied protein-protein docking using the available structures of the interacting partners and their biological units in order to predict the binding residues for these interactions.

Mutations in the same gene can affect different phenotypic traits (pleiotropy). In this context, the number of interactions and interfaces in a protein is key to understand pleiotropic effects in disease genes. Recent studies show that SAVs located at distinct protein-protein interfaces of the same protein are prone to produce different disease phenotypes [20,26,27]. Moreover, it has been demonstrated that one-third of the SAVs produce an ‘edgetic’ effect, by impairing only a subset of the interactions [20].

**Figure 5:**
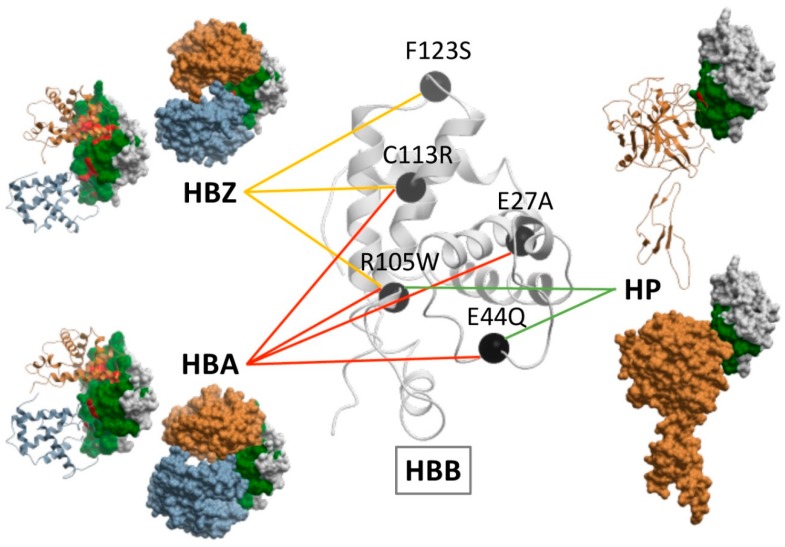
Estimation of the effect of disease-causing variants in the HBB interaction network based on experimentally solved complex structures. HBB is represented in white ribbon, with selected pathogenic variants in black, linked to the protein-protein interactions in whose interface they are located. The structures of such interactions are represented, showing HBB in white skin with interface rim residues (in green) and interface core residues (in red), and HBB partners (HBZ, HBA, HP) either as ribbon or skin in two different views.

In this line, structural analysis of the case example hemoglobin subunit beta (HBB) showed that the same variant could affect the interaction with different partner proteins if their interface patches are the same, and different variants could perturb different partner proteins if these have distinct interface patches. Figure 5 shows some of the pathogenic mutations found in HBB as well as the interaction they impair. For instance, F123S only affects the interaction between HBB and hemoglobin subunit zeta (HBZ); E27A perturbs the interaction between HBB and hemoglobin subunit alpha (HBA); E44Q hampers the interactions between HBB and both HBA and haptoglobin (HP); C113R affects the interactions of HBB with HBA and HBZ, same as R105W, which also hinders the interaction between HBB and HP.

The extensive analysis of protein interface residues shown here, combining complex structures and docking predictions, demonstrate that pathogenic variants are more likely to be located at the interface rather than at the non-interacting surface. More precisely, we found that they are more probable to occur at the interface core region rather than at the rim, in agreement with previous studies [4,17,18,28]. On the contrary, neutral variants occur significantly more often in the interface rim as well as in the non-interacting surface, as compared with the interface core region. Furthermore, the residues that contribute the most to the binding free energy of the protein-protein complex (hot-spots) are more likely to be located at the interface core. This is in line with previous studies [18,21,29], which revealed that hot-spot residues are not equally distributed among interface regions, but they tend to be clustered within the interface core. Thus, this core region is critical for the stabilization of PPIs; this is reflected in the fact that core residues show a higher level of conservation and coevolution among homologous proteins as compared to those in the rim [23,30]. This energetical relevance of the core region also explains why protein sequence variants are not as likely to be tolerated there as in the interface rim or the non-interacting surface [18]. We found that arginine, tryptophan and tyrosine are over-represented among disease-causing mutated residues. This was consistent with previous studies reporting that the most frequent hot-spot residues are tryptophan (21%), arginine (13.3%) and tyrosine (12.3%) [31,32,33]. Indeed, arginine mutations in interface core residues are not likely to be tolerated and tend to have a profound effect in phenotype [31,32].

The present study has some limitations, such as the low availability of 3D protein structures or the global consideration in the analysis of both transient and permanent PPIs, which are known to show very different mechanistic, structural and energetic properties. Despite these limitations, this study shows that the structural characterization of PPIs and the analysis of the location of pathogenic and neutral variants, together with the identification of the interface residues that are more prone to be mutated and lead to disease, can provide novel information on disease-causing variants. This can be useful in order to characterize protein sequence variants in future studies, interpret them at the molecular level, improve the accuracy of pathogenicity predictors on new mutations, and help to advance toward precision medicine by helping clinicians to provide adequate diagnosis and treatments.

Further studies with more docking simulations will need to be undertaken. For partners with 3D structures split in different PDBs, a template could be used to model the missing amino acids and join the distinct protein fragments in a correct global 3D structure. Moreover, if these PDBs contain at least one complete domain, docking simulations could be done at the domain level. Homology models could be generated for those proteins without available 3D structure, so that docking can be run afterwards in order to find a possible protein-protein interface. This would help to achieve a better understanding of disease at molecular level since more PPIs could be characterized and more disease-causing and neutral variants could be mapped on the structural models.

## 4. Materials and Methods

### 4.1. Protein Interaction and Mutational Data

Human PPI data and structural information for both protein complexes and their individual components were retrieved from Interactome3D [25]. In this database, human protein-protein interaction data is generated by integrating information available from nine major public PPI databases: Intact [34], MINT [35], DIP [36], MPIDB [37], MatrixDb [38], InnateDb [39], BioGRID [40], BIND [41] and HPRD [42]. The Interactome3D database also provided the experimentally solved structures of protein-protein complexes, when they were available in the Protein Data Bank (PDB) [43].

Human pathogenic mutations were compiled by pooling variants obtained from UniProt [44], selecting SAVs labelled as “Disease” in the downloadable file humsavar.txt (therefore not including “Polymorphism” or “Unclassified” variants), as well as from the Human Gene Mutation Database (HGMD) [45], containing both missense and nonsense variants (Table S1). For neutral variants (Table S1), we used the homology-based model described in Riera et al. [15,46,47], where variants were obtained from a multiple sequence alignment (MSA) for each protein family and corresponded to mismatches between the human protein and its close homologs (more than 95% sequence identity with respect to the human protein sequence).

### 4.2. Interacting Proteins Analysis

As a further analysis, protein structures involved in each interaction were characterized in more detail regarding sequence identity, structural coverage, domains, and biological assembly (Table S2). Sequence identity and structural coverage were calculated using the UniProt canonical sequence as a reference. Missing loops were not considered in the structural coverage calculations (as opposed to the structural coverage value given by Interactome3D, which includes the missing loops). To identify the protein domains, HMMER3 [48] was used to search against Pfam database [49], based on the canonical sequence. Based on the structural coverage, PPIs could be defined depending on whether the interacting proteins had: (i) global structural coverage greater than 80% in a single PDB file, (ii) global structural coverage < 80% and at least one domain with more than 80% structural coverage, and (iii) global or domain structural coverage < 80% (Table S2).

### 4.3. Experimental Protein-Protein Interfaces

Protein-protein complex structures, when available, were retrieved from PDB based on Interactome3D information. Protein-protein interfaces were defined in a similar way as previously described [50]. Prior to the interface calculation, the sequence and numbering of the PDB structures were extracted and aligned with the corresponding canonical sequence fetched from UniProt database to ensure a correct residue numbering.

Residues were defined as buried if they had relative Accessible Surface Area in the uncomplexed structure (rASA_u_) < 0.1, or surface if they had rASA_u_ ≥ 0.1. Surface residues were classified as interface residues when the difference in rASA between the uncomplexed and complexed form (rASA_u_-rASA_c_) was > 0, or non-interface surface otherwise. Interface residues were further divided into core and rim. Core was formed by interface residues that were buried in the complex (rASA_c_ < 0.1), and rim was formed by interface residues that remained exposed in the complex (rASA_c_ > 0.1). The value rASA was computed as the ratio between the Accessible Surface Area (ASA) of a given residue, and the ASA of the corresponding residue type in the extended conformation of the Gly-X-Gly peptide. All (ASA) calculations were done with ICM-Browser (http://www.molsoft.com).

### 4.4. Predicted Protein-Protein Interfaces

For selected protein-protein interactions without available protein complex structure, we applied a computational procedure to estimate the interface residues. For this, the uncomplexed structures were retrieved from PDB, considering the oligomeric state as defined in the biological unit in the PDB. In this work, ab initio protein-protein docking was used to model the PPI when both proteins forming the complex had more than 80% structural coverage.

First, the sequence and numbering of the PDB structures were extracted and aligned with the corresponding canonical sequence fetched from UniProt database, to ensure a correct residue numbering. Then, docking simulations were run with the Fast Fourier Transform (FFT)-based program FTDock 2.0 [51], and the resulting 10,000 rigid-body orientations were rescored by pyDock scoring function, which includes electrostatics, desolvation energy, and a limited van der Waals contribution [52].

From the resulting docking poses, a normalized interface propensity (NIP) was obtained per residue with the built-in *patch* module in pyDock, implementing the pyDockNIP algorithm [53]. A normalized interface propensity (NIP) value of one indicates that the corresponding residue is involved in all predicted interfaces of the 100 lowest energy docking solutions, while a value of zero means that it appears as expected by random. On the other hand, a negative NIP value implies that the residue appears at the low-energy docking interfaces less often than expected by random [53]. Usually, residues with NIP ≥ 0.2 are considered as hot-spot residues when using FTDock but given the large size of the proteins analyzed here, we used a cutoff of NIP ≥ 0.1 to define the predicted hot-spot residues. These constituted the predicted interface core residues. Then, predicted interface rim residues were built by surface residues located within 10 Å distance from the predicted hot-spot (core) residues [28].

### 4.5. Energetic Characterization of Protein-Protein Interfaces

The energetic characterization of protein-protein interfaces was performed with the pyDock *bindEy* and *resEnergy* modules. The *bindEy* module computes the total binding energy for a given protein-protein interaction, based on the complex structure or a model. The *resEnergy* module calculates the contribution of each individual protein residue to the binding energy for a given protein-protein complex structure.

### 4.6. Statistical Analysis

The statistical analyses were performed using version 3.4.4 of the R statistical package [54]. The probability of observing a protein sequence variant in the protein region *i* is calculated as shown in Equation (1), where *n_i_* is the number of variants observed in the protein region *i*, and *N_i_* is the total number of residues in that region. The likelihood of a variant to be in region *i* rather than in region j in the protein was expressed then in terms of odds ratio (OR*_ij_*) (Equation (2)). The χ^2^ test was used to compare the observed number of variants in each region with the expected one if variants were distributed according to the number of residues in the different regions. A two-tailed *p*-value < 0.05 indicated statistical significance of the preference for variants to be in one region over another. Bonferroni correction was used to adjust *p*-value for multiple comparisons.

(1) $$x_{i}=\frac{n_{i}}{N_{i}}$$

(2) $$OR_{ij}=\frac{x_{i}/\left(1-x_{i}\right)}{x_{j}/\left(1-x_{j}\right)}$$

The amino acid substitution susceptibility to disease-causing variants or neutral ones at protein interfaces was calculated, and a two-tailed *p*-value < 0.05 implied statistical significance according to an “*N-1*” χ^2^ test.

## Figures and Tables

**Table 1:** Distribution of residues along the different protein regions and odds ratio for disease-causing and neutral variants.

***Disease-causing SAVs***									
**Region**	**All Residues ^1^**	**Observed ^2^**	**Expected ^3^**	**O/E ^4^**	**Regions**	**OR ^5^**	**95% C.I.**	***p*-value**	**Adjusted *p*-Value**
Buried	6019	1842	1.548.96	1.19	Buried versus Surface	2.05	1.83–2.28	<0.00001	<0.00001
Surface	3118	552	802.40	0.69	Core versus Buried	0.94	0.82–1.09	0.441	1
Rim	916	151	235.73	0.64	Core versus Rim	2.11	1.69–2.64	<0.00001	<0.00001
Core	1146	337	294.92	1.14	Core versus Surface	1.94	1.65–2.27	<0.00001	<0.00001
Total	11199	2882			Rim versus Surface	0.92	0.75–1.12	0.428	1
					Rim versus Buried	0.45	0.37–0.54	<0.00001	<0.00001
					Interface versus Surface	1.44	1.25–1.54	<0.00001	<0.00001
***Neutral SAVs***									
**Region**	**All Residues ^1^**	**Observed ^2^**	**Expected ^3^**	**O/E ^4^**	**Regions**	**OR ^5^**	**95% C.I.**	***p*-Value**	**Adjusted *p*-Value**
Buried	6019	524	834.14	0.63	Buried versus Surface	0.29	0.25–0.33	<0.00001	<0.00001
Surface	3118	767	432.10	1.78	Core versus Buried	0.82	0.63–1.04	0.105	0.738
Rim	916	178	126.94	1.40	Core versus Rim	0.32	0.24–0.43	<0.00001	<0.00001
Core	1146	83	158.82	0.52	Core versus Surface	0.24	0.19–0.30	<0.00001	<0.00001
Total	11199	1552			Rim versus Surface	0.74	0.61–0.89	0.001187	0.008209
					Rim versus Buried	2.53	2.08–3.06	<0.00001	<0.00001
					Interface versus Surface	0.44	0.38–0.52	<0.00001	<0.00001

^1^ Total number of residues in each protein region. ^2^ Observed number of residues involving pathogenic (or neutral) variants in each protein region. ^3^ Expected number of residues involving pathogenic (or neutral) variants in each protein region, according to a random distribution based on the total number of residues. ^4^ Ratio of observed to expected residues involving pathogenic (or neutral) variants in each protein region. ^5^ Odds ratio for different possibilities is calculated with a 95% confidence interval and a *p*-value for a two-tailed Fisher’s exact test. This *p*-value is adjusted using Bonferroni correction. A *p*-value < 0.05 is considered indicative of statistical significance.

**Table 2:** Distribution of all interface residues and those energetically relevant for the interaction.

Interface Region	All Residues ^1^	Observed Low-Energy Residues ^2^	Expected Low-Energy Residues ^3^	O/E ^4^
Rim	916	201	298.08	0.67
Core	1146	470	372.92	1.26
Total	2062	671		

^1^ Total number of residues in the set of PPIs analyzed here in each interface region (core and rim). ^2^ Residues with binding energy < −2 a.u., as calculated by pyDock, in each interface region. ^3^ Expected number of low binding energy residues in each interface region according to a random distribution based on the total number of residues. ^4^ Ratio of observed to expected residues.

**Table 3:** Docking-based characterization of HADHA mutations.

UniProt ^1^ (Partner)	Neutral Mutations		Pathogenic Mutations ^2^	
	Core ^3^	Rim ^3^	Core ^3^	Rim ^3^
O95166	D398G	A396G, K406R	-	R399 *, V412L
P60520	-	D398G, K406R	-	R399 *, V412L
Q14164	D398G	A396G, K406R, K519R, A596V, S654N, K734Q	-	*Q358K*, R399 *, V412L, R610G, Y740 *
Q99714	D398G, S654N	A396G, K406R, A596V, R645S, R645N, L661I, K734Q	-	R399 *, V412L, R610G, Y740 *
Q9GZQ8	D398G	V52I, V526I, N142S, L221I, E223T, I237M, A396G, K406R	-	R399 *, V412L
Q9H0R8	D398G	N142S, L221I, E223T, I237M, A396G, K406R, S654N, L661I	-	R399 *, V412L

^1^ UniProt code of the corresponding interacting partner. ^2^ The symbol “*” indicates stop codon. Mutations R399*, Y740* and V412L (in bold) are associated with mitochondrial trifunctional protein deficiency. Mutation Q358K (in italics) is associated to haemolysis, elevated liver enzymes, and low platelets. Mutation R610G is associated to long-chain 3-hydroxyacyl-CoA dehydrogenase deficiency. ^3^ Interface core and rim estimated from the docking calculations.

## Supplementary Materials

Supplementary Materials can be found at https://www.mdpi.com/1422-0067/20/7/1583/s1.

## Abbreviations

NGS	Next-generation sequencing
SNV	Single nucleotide variant
SAV	Single amino acid variant
PPI	Protein-protein interaction
HBB	Hemoglobin subunit beta
HBZ	Hemoglobin subunit zeta
HBA	Hemoglobin subunit alpha
HP	Haptoglobin
PDB	Protein Data Bank
MSA	Multiple Sequence Alignment
ASA	Accessible Surface Area
NIP	Normalized Interface Propensity
HADHA	Hydroxyacyl-CoA dehydrogenase trifunctional multienzyme complex subunit α
