# Supplementary material for: Structural and Computational Characterization of Disease-Related Mutations Involved in Protein-Protein Interfaces
Source: Int J Mol Sci. 2019 Mar 29;20(7):1583. doi: 10.3390/ijms20071583 (PMC6479360; doi:10.3390/ijms20071583)
Supplement: Supplementary file 1 [file ijms-20-01583-s001.zip › Navio_et_al_Table_S1.pdf]

Table S1. Proteins associated to diseases detected in newborn screening, with known pathological and neutral mutations

Gene	UniProt	Disease [1]	Neutral mutations	Disease mutations
ABCD4	O14678	Methylmalonic aciduria and homocystinuria (cblJ type)	80	2
ACADM	P11310	Medium chain acyl CoA dehydrogenase deficiency	43	119
ACADS	P16219	Small chain Acyl-CoA-dehydrogenase deficiency<br>Ethylmalonic aciduria	51	66
ACADSB	P45954	2-Methylbutyryl-CoA dehydrogenase deficiency	34	10
ACADVL	P49748	Very long chain acyl-CoA dehydrogenase deficiency<br>Carnitine palmitoyltransferase 2 deficiency	114	153
ACAT1	P24752	Acetoacetyl-CoA-thiolase deficiency<br>Alpha-methylacetoacetic aciduria<br>3-ketothiolase deficiency	22	41
ACSF3	Q4G176	Malonic & methylmalonic aciduria<br>Methylmalonic acidaemia	47	15
ARG1	P05089	Argininemia / Arginase I deficiency	46	26
ASL	P04424	Argininosuccinic aciduria<br>Argininosuccinate lyase deficiency	120	111
ASS1	P00966	Citrullinaemia	152	84
BCKDHA	P12694	Maple syrup urine disease	43	64
BCKDHB	P21953	Maple syrup urine disease	47	64
BTD	P43251	Biotinidase deficiency	65	180
CBS	P35520	Homocystinuria<br>Myelomeningocele	39	130
CFTR	P13569	Cystic fibrosis<br>Pancreatitis chronic<br>Hypertrypsinaemia, neonatal<br>Asthma<br>Chronic pulmonary disease<br>Congenital absence of vas deferens<br>Primary sclerosing colangitis<br>Bronchiectasis	225	963
CPT1A	P50416	Carnitine palmitoyltransferase 1 deficiency	89	34
CPT1B	Q92523	Carnitine palmitoyltransferase 1 deficiency<br>Autism spectrum disorder<br>Obesity	83	2
CPT1C	Q8TCG5	Carnitine palmitoyltransferase 1 deficiency	56	1
CPT2	P23786	Carnitine palmitoyltransferase 2 deficiency<br>Hypercholesterolaemia<br>Muscular dystrophy, limb-girdle	74	73
CYP11B1	P15538	Congenital Adrenal hyperplasia	64	88
CYP17A1	P05093	Congenital Adrenal hyperplasia<br>Pseudohermaphroditism	29	74
CYP21A2	P08686	Congenital Adrenal hyperplasia<br>Premature pubarche	81	170
DBT	P11182	Maple syrup urine disease	38	39
ETFA	P13804	Glutaric acidaemia type 2	116	17
ETFB	P38117	Glutaric acidaemia type 2	83	7
ETFDH	Q16134	Glutaric acidaemia type 2<br>Acyl-CoA dehydrogenation deficiency<br>Coenzyme Q10 deficiency<br>Lipid storage myopathy<br>Subacute myopathy	128	130
FAH	P16930	Tyrosinaemia type 1	39	57
FCGR2A	P12318	Cystic fibrosis<br>Lupus nephritis	14	3
GALT	P07902	Galactosaemia	33	249
GCDH	Q92947	Glutaric acidaemia type I	78	162
HADHA	P40939	Long-chain 3-hydroxyacyl-CoA dehydrogenase deficiency<br>Mitochondrial trifunctional protein deficiency<br>Rhabdomyolysis	116	31
HADHB	P55084	Mitochondrial trifunctional protein deficiency<br>Charcot-Marie-Tooth disease<br>Hypoparathyroidism, infantile-onset<br>Peripheral polyneuropathy	70	35
HBB	P68871	Thalassaemia beta<br>Sickle cell anaemia<br>Erythrocytosis<br>Dyspnea, progressive	31	389
HCFC1	P51610	Methylmalonic acidemia with homocystinuria<br>Dysmorphic features<br>Hypospadias<br>Intellectual disability (nonsyndromic/X-linked)	215	14

		Mental retardation (X-linked)		
HMGCL	P35914	HMG-CoA lyase deficiency<br>3-hydroxy-3-methylglutaric aciduria	27	30
HPD	P32754	Tyrosinaemia<br>Hawkinsinuria	34	8
HSD3B2	P26439	Congenital adrenal hyperplasia<br>Hypospadias / Idiopathic hypospadias<br>Pseudohermaphroditism	48	46
IVD	P26440	Isovaleric acidaemia<br>Complex I deficiency	73	50
LMBRD1	Q9NUN5	Methylmalonic aciduria and homocystinuria (cblF type)	202	0
MCCC1	Q96RQ3	3-methylcrotonyl-CoA carboxylase deficiency	63	60
MCCC2	Q9HCC0	3-methylcrotonyl-CoA carboxylase deficiency<br>Complex I deficiency	36	85
MLYCD	O95822	Malonyl-CoA decarboxylase deficiency	51	17
MMAA	Q8IVH4	Methylmalonic acidaemia/aciduria (cblA type)	49	31
MMAB	Q96EY8	Methylmalonic acidemia (cblB type)	8	21
MMACHC	Q9Y4U1	Methylmalonic acidemia (cblC type)<br>Homocystinuria (cblC type)	38	43
MMADHC	Q9H3L0	Methylmalonic aciduria (cblD type)<br>Homocystinuria (cblD type)	93	8
MTHFR	P42898	Homocystinuria<br>Critical congenital Heart Disease<br>Neural tube defect<br>Preeclampsia<br>Inborn errors of metabolism<br>Cleft lip and/or palate	51	94
MUT	P22033	Methylmalonic acidemia	224	224
PAH	P00439	Classic phenylketonuria<br>Benign hyperphenylalaninaemia	86	582
PAX8	Q06710	Primary congenital hypothyroidism	136	27
PCCA	P05165	Propionic acidaemia	101	51
PCCB	P05166	Propionic acidaemia	217	63
SLC22A5	O76082	Carnitine deficiency	70	79
SLC25A13	Q9UJS0	Citrullinemia, type 2	355	64
SLC25A20	O43772	Carnitine-acylcarnitine translocase deficiency	87	19
TAT	P17735	Tyrosinaemia type 2	50	15
TGFB1	P01137	Cystic fibrosis<br>Osteoporosis / Ostosclerosis<br>Camurati-Engelmann<br>Cleft-lip	65	16
TSHR	P16473	Primary congenital hypothyroidism	73	107

[1] Disease detected in newborn screening (first line) and other associated diseases.
