# Supplementary material for: Structural and Computational Characterization of Disease-Related Mutations Involved in Protein-Protein Interfaces
Source: Int J Mol Sci. 2019 Mar 29;20(7):1583. doi: 10.3390/ijms20071583 (PMC6479360; doi:10.3390/ijms20071583)
Supplement: Supplementary file 1 [file ijms-20-01583-s001.zip › Navio_et_al_Table_S2.pdf]

Table S2. Structural data on analysed proteins and their interacting partners: location of mutations in protein-protein interfaces

		Individual				Complex	Neutral mutations [7]		Disease mutations [7]	
**Gene [1]**	**UniProt [1]**	**PDB [2]**	**Glob. Cov. [3]**	**Domain Cov. [4]**	**Biological Unit [5]**	**PDB [6]**	**CORE**	**RIM**	**CORE**	**RIM**
**ABCD4**	**O14678**	(2hyd_A)	96	98/100	Homo 2-mer	-	-	-	-	-
HSPB2	Q16082	(3j07_A)	95	100	Homo 24-mer	-	-	-	-	-
PEA15	Q15121	1n3k_A	100	100	Monomer	-			-	-
XRCC6	P12956	1jeq_A	90	100/78/97/100	Hetero 2-mer	-	-	-	-	-
SARAF	Q96BY9	-	-	-	-	-	-	-	-	-
DLEU1	O43261	-	-	-	-	-	-	-	-	-
All PPIs [8]							**-**	**-**	**-**	**-**
**ACADM**	**P11310**	4p13_A	92	100/100/100/100	Homo 4-mer	4p13	1	3	9	11
USP50	Q70EL3	(2hd5_A)	88	100/100	Monomer	-	-	-	-	-
ETFB	P38117	1efv_B	99	100	Hetero 2-mer	2a1t	0	0	3	2
ETFA	P13804	1efv_A	94	100/100	Hetero 2-mer	2a1t	0	0	1	4
USP20	Q9Y2K6	5z4i_A	11	0/0/0/100	Monomer	-	-	-	-	-
All PPIs [8]							***0***	***3***	***10***	***12***
**ACADS**	**P16219**	2vig_E	92	100/100/100/100	Homo 4-mer	2vig	2	0	8	3
IRF7	Q92985	2o61_A	65	100/59	Homo 2-mer	-	-	-	-	-
All PPIs [8]							***2***	***0***	***8***	***1***
**ACADSB**	**P45954**	2jif_A	88	100/100/100/100	Homo 4-mer	2jif	1	2	1	1
USP19	O94966	1wh0_A	10	100/0/0/0/15	Monomer	-	-	-	-	-
All PPIs [8]							***1***	***1***	***1***	***1***
**ACADVL**	**P49748**	2uxw_A	87	100/100/100/100	Homo 2-mer	2uxw	5	8	13	7
SOCS3	O14543	(2c9w_A)	85	100	Monomer	-	-	-	-	-
All PPIs [8]							***5***	***8***	***13***	***7***
**ACAT1**	**P24752**	2ib8_D	92	100/100/100	Homo 4-mer	2ib8	0	2	2	2
PEX7	O00628	(3w15_A)	93	93	Hetero 3-mer	-	-	-	-	-
EIF1B	O60739	(2if1_A)	100	100	Monomer	-	-	-	-	-
MPG	P29372	1f6o_A	71	97	Monomer	-	-	-	-	-
HLA-C	P30504	1im9_E	75	0/100/100	Hetero 5-mer	-	-	-	-	-
GH1	P01241	1hwh_A	85	88	Hetero 2-mer	-	-	-	-	-
TNIK	Q9UKE5	5d7a_A	21	96/96/0	Monomer	-	-	-	-	-
HLA-B	P30480	4u1m_A	76	0/100/100	Hetero 2-mer	-	-	-	-	-
MCC	P23508	-	-	-	-	-	-	-	-	-
All PPIs [8]							***0***	***2***	***1***	***2***
**ACSF3**	**Q4G176**	(3nyq_A)	89	100/93	Monomer	N/A				
MATN4	O95460	(1ck4_A) (1mjn_A)	30	0/0/100/100/100/100/0/0/0/0	Monomer	-	-	-	-	-
TRIM27	P14373	(4cg4_A) (2cvs_A)	71	0/100/100/0/100/0	Homo 2-mer	-	-	-	-	-
PPP1R13B	Q96KQ4	(1ycs_B)	18	100/100/100/100/100/100	Monomer	-	-	-	-	-
KRT40	Q6A162	-	-	-	-	-	-	-	-	-
**ARG1**	**P05089**	3gmz_A	98	100	Homo 3-mer	2aeb	0	2	2	0
FLOT1	O75955	(1win_A)	32	77/0	Monomer	-	-	-	-	-
USP53	Q70EK8	-	-	-	-	-	-	-	-	-
All PPIs [8]							***0***	***2***	***2***	***0***

**ASL**	**P04424**	1k62_B	99	100/100	Homo 4-mer	1k62	6	8	26	12
HMOX1	P09601	(1wov_A)	81	100	Homo 2-mer	-	-	-	-	-
TRIM3	O75382	(1jm7_A)	10	0/0/0/0/0/100/<br>0/0/100/100/1<br>00/100/100/0	Homo 2-mer	-	-	-	-	-
WDYHV1	Q96HA8	4w79_A	98	100	Monomer	-	-	-	-	-
MCMBP	Q9BTE3	4kg9_B	1	1	Monomer	-	-	-	-	-
USP53	Q70EK8	-	-	-	-	-	-	-	-	-
***All PPIs* [8]**							***6***	***7***	***26***	***5***
**ASS1**	**P00966**	2nz2_A	98	99	Homo 4-mer	2nz2	11	12	13	6
ARAF	P10398	(3lb7_A)<br>(1faq_A)	45	100/100/100/<br>100/0	Homo 2-mer<br>Monomer	-	-	-	-	-
PTPN1	P18031	(1lar_A)	82	100	Monomer	-	-	-	-	-
TERF2	Q15554	3bu8_B	38	0/100/0	Homo 4-mer	-	-	-	-	-
USP4	Q13107	2y6e_B<br>(3jyu_A)	35	100/52/81/100	Homo 6-mer<br>Homo 2-mer	-	-	-	-	-
TERF1	P54274	3bqo_A<br>1ity_A	46	100/97	Homo 2-mer<br>Monomer	-	-	-	-	-
MLH1	P40692	(3na3_A)<br>3rbn_A	44	100/100/100/<br>94	Homo 2-mer	-	-	-	-	-
ASCC2	Q9H1I8	2di0_A	9	100	Monomer	-	-	-	-	-
JAK2	O60674	4gmy_A<br>4z32_A<br>4fvr_A	39	100/100/100/<br>100/100	Monomer	-	-	-	-	-
CDR2	Q01850	-	-	-	-	-	-	-	-	-
AKTIP	Q9H8T0	-	-	-	-	-	-	-	-	-
***All PPIs* [8]**							***11***	***12***	***13***	***3***
**BCKDHA**	**P12694**	1olx_A	88	98	Hetero 4-mer	2bfd	1	1	3	3
BCKDHB	P21953	2bev_B	86	100/100	Hetero 4-mer	2bfd	1	1	6	3
BCKDK	O14874	(1gkz_A)	83	100/100	Monomer	-	-	-	-	-
***All PPIs* [8]**							***2***	***2***	***9***	***3***
**BCKDHB**	**P21953**	2bev_B	86	100/100	Hetero 4-mer	2bfd	0	0	6	2
BCKDHA	P12694	1olx_A	88	98	Hetero 4-mer	2bfd	0	0	9	2
***All PPIs* [8]**							***0***	***0***	***14***	***1***
**BTD**	**P43251**	(4cyf_A)	90	100	Monomer	N/A				
**CBS**	**P35520**	4l3v_A	90	100/100	Homo 2-mer	4l28	1	5	6	5
PRKAR1A	P10644	(4din_B)	96	100/100/100	Monomer	-	-	-	-	-
PIAS1	O75925	(4mvt_A)	43	100/100	Monomer	-	-	-	-	-
EHHADH	Q08426	(3zwc_A)	100	100/100/100/<br>100/100	Monomer	-	-	-	-	-
UBASH3A	P57075	(3d4i_A)<br>(1gri_A)	42	100/100	Homo 2-mer	-	-	-	-	-
FXR2	P51116	(4qw2_A)<br>(2qnd_A)	29	100/0/4/100	Homo 2-mer	-	-	-	-	-
CBX4	O00257	2k28_A	11	0/100	Monomer	-	-	-	-	-
PRKAG1	P54619	5ezv_E	91	100/100/100	Hetero 3-mer	-	-	-	-	-
ZDHHC17	Q8IUH5	3eu9_A	34	100/0/100/100<br>/100/100/100/<br>100/100	Homo 6-mer	-	-	-	-	-
WDYHV1	Q96HA8	4w79_A	98	100	Monomer	-	-	-	-	-
PSMA1	P25786	4r3o_F	90	100/100	Hetero 28-mer	-	-	-	-	-
PIAS3	Q9Y6X2	4mvt_A	41	100/82	Monomer	-	-	-	-	-
UGP2	Q16851	3r2w_D	92	95	Homo 8-mer	-	-	-	-	-
VTA1	Q9NP79	2lxm_A	53	100	Hetero 2-mer	-	-	-	-	-
RANBP9	Q96S59	5jiu_B	29	100/0/0	Homo 2-mer	-	-	-	-	-
COL4A3BP	Q9Y5P4	2e3p_A<br>4hhv_A	38	100/100/100	Homo 2-mer	-	-	-	-	-
PIN1	Q13526	1nmv_A	100	100/100/100	Monomer	-	-	-	-	-
HID1	Q8IV36	-	-	-	-	-	-	-	-	-
***All PPIs* [8]**							***1***	***5***	***6***	***4***

**CFTR**	**P13569**	5uak_A	77	100/91/6/100	Homo 5-mer<br>Monomer	1xmi	4	5	26	30
LPAR2	Q9HBW0	(4z35_A)	87	100/100	Monomer	-	-	-	-	-
CANX	P27824	(1jhn_A)	67	0/100	Monomer	-	-	-	-	-
DERL1	Q9BUN8	5glf_B	5	0	Homo 4-mer	-	-	-	-	-
BCAP31	P51572	4jzl_C	25	24	Homo 2-mer	-	-	-	-	-
SLC9A3R1	O14745	2krg_A<br>2m0u_A	60	100/100/100/<br>100/100	Monomer	-	-	-	-	-
FLNA	P21333	(4b7l_A)<br>1wlh_A)<br>2j3s_A<br>2k3t_A<br>4m9p_A<br>2k7p_A<br>3rgh_A<br>3cnk_A	13	100/3/100/<br>100/100/0/0/0/<br>0/100/0/100/<br>100/100/100/<br>100/0/100/100<br>/0/100/95/100/<br>100	Monomer<br>Homo 2-mer	-	-	-	-	-
PPP2R1A	P30153	1b3u_B	100	100/100	Monomer	-	-	-	-	-
YWHAZ	P63104	4zdr_A	96	99	Homo 2-mer	-	-	-	-	-
GOPC	Q9HD26	4q6h_A	19	100	Monomer	-	-	-	-	-
PRKAG1	P54619	5ezv_E	91	100/100/100	Hetero 3-mer	-	-	-	-	-
GORASP2	Q9H8Y8	4edj_A	45	100/100	Monomer	-	-	-	-	-
PDZD3	Q86UT5	2v90_A	17	0/0/100/0/0	Homo 6-mer	-	-	-	-	-
YWHAG	P61981	4j6s_B	97	100	Monomer	-	-	-	-	-
CFTR	P13569	5uak_A	77	100/91/6/100	Monomer<br>Homo 5-mer	-	-	-	-	-
KCNN4	O15554	-	-	-	-	-	-	-	-	-
KRT8	P05787	-	-	-	-	-	-	-	-	-
ANO1	Q5XXA6	-	-	-	-	-	-	-	-	-
SLC26A8	Q96RN1	-	-	-	-	-	-	-	-	-
RNF5	Q99942	-	-	-	-	-	-	-	-	-
DERL1	Q9BUN8	-	-	-	-	-	-	-	-	-
All PPIs [8]							***1***	***3***	***21***	***16***
**CPT1A**	**P50416**	(2fy5_A)	77	100/0	Monomer	N/A				
CLIC1	O00299	3uvh_A	96	92/100/100	Homo 2-mer	-	-	-	-	-
CACYBP	Q9HB71	1x5m_A<br>2jtt_C<br>(1ysm_A)	56	69/100/55	Monomer	-	-	-	-	-
KBTBD7	Q8WVZ9	-	-	-	-	-	-	-	-	-
**CPT1B**	**Q92523**	(1nm8_A)	77	0/100	Monomer	N/A				
TEX44	Q53QW1	-	-	-	-	-	-	-	-	-
**CPT1C**	**Q8TCG5**	2m76_A	6	100/0	Monomer	N/A				
**CPT2**	**P23786**	(2deb_A)	95	100	Monomer	N/A				
**CYP11B1**	**P15538**	(4dvq_A)	93	100	Homo 6-mer	-	-	-	-	-
**CYP17A1**	**P05093**	5irv_C	93	100	Monomer	N/A				
CDH1	P12830	(3q2v_A)<br>(1op4_A)	61	100/0/100/100<br>/100/100/100	Monomer	-	-	-	-	-
TIGD7	Q6NT04	(1hlv_A)	24	100/88/0	Monomer	-	-	-	-	-
APC	P25054	3nmz_A<br>1deb_B<br>1m5i_A<br>1th1_C<br>1emu_B<br>2rqu_B	14	0/0/0/0/0/0/0/<br>0/0/100/0/100/<br>2/0/0/50/76/93	Monomer	-	-	-	-	-
PTPRJ	Q12913	2nz6_A	22	93	Monomer	-	-	-	-	-
IGF1R	P08069	1igr_A<br>1p4o_B	34	100/100/100/<br>100/100	Monomer	-	-	-	-	-
CDO1	Q16878	2ic1_A	92	97	Monomer	-	-	-	-	-
CHEK2	O96017	3i6u_A	72	91/100/91/91	Homo 2-mer	-	-	-	-	-
HSP90AB1	P08238	5fwk_B	87	100/100/87	Monomer<br>Hetero 4-mer	-	-	-	-	-
BARX1	Q9HBU1	2dmt_A	31	100	Monomer	-	-	-	-	-
STK11	Q15831	2wtk_C	66	99/100/99	Monomer<br>Hetero 3-mer	-	-	-	-	-
ERBB2	P04626	3n85_A	48	99/100/99/92/	Hetero 9-mer	-	-	-	-	-

		3pp0_B		100/97	Monomer					
ATP5E	P56381	(2v7q_I)	92	96	Homo 3-mer	-	-	-	-	-
RPAIN	Q86UA6	-	-	-	-	-	-	-	-	-
**CYP21A2**	**P08686**	4y8w_C	89	96	Monomer	N/A				
**DBT**	**P11182**	(2ii3_A)<br>2coo_A<br>1k8m_A	48	100/100/100	Homo 24-mer<br>Monomer	-	-	-	-	-
DLD	P09622	1zmd_A	93	100/100/100/<br>100/100/100/<br>100	Homo 2-mer	-	-	-	-	-
USP16	Q9Y5T5	2i50_A	15	100/0/0	Monomer	-	-	-	-	-
ADI1	Q9BV57	4qgn_A	99	100/100	Monomer	-	-	-	-	-
**ETFA**	**P13804**	1efv_A	94	100/100	Hetero 2-mer	N/A				
ETFB	P38117	1efv_B	99	100	Hetero 2-mer	1efv	5	6	2	1
ACADM	P11310	4p13_A	92	100/100100/<br>100	Homo 4-mer	2a1t	0	0	0	2
HSPB2	Q16082	(3j07_A)	94	100	Homo 24-mer	-	-	-	-	-
IKBKE	Q14164	(4im0_A)	90	100/100	Homo 2-mer	-	-	-	-	-
FTSJ1	Q9UET6	(1ej0_A)	55	100	Monomer	-	-	-	-	-
EIF1B	O60739	(2if1_A)	100	100	Monomer	-	-	-	-	-
ELF3	P78545	3jtg_A<br>2e8p_A	25	100/100	Monomer	-	-	-	-	-
TRAF6	Q9Y4K3	3hcs_A	30	12/100/100/<br>100/100/100/<br>100/	Homo 2-mer	-	-	-	-	-
CDC42	P60953	5c2j_B	100	100/100/100	Hetero 2-mer	-	-	-	-	-
PSEN1	P49768	5fn5_B	48	54	Monomer	-	-	-	-	-
MPG	P29372	1f6o_A	71	97	Monomer	-	-	-	-	-
PRKAB1	Q9Y478	5ezv_D	69	100/100	Hetero 3-mer	-	-	-	-	-
MCC	P23508	-	-	-	-	-	-	-	-	-
All PPIs [8]							***5***	***6***	***2***	***1***
**ETFB**	**P38117**	1efv_B	99	100	Hetero 2-mer	N/A				
ETFA	P13804	1efv_A	94	100/100	Hetero 2-mer	1efv	12	10	1	0
ACADM	P11310	4p13_A	92	100/100/100/<br>100	Homo 4-mer	2a1t	0	2	0	1
IKBKE	Q14164	(4im0_A)	90	100/100	Homo 2-mer	-	-	-	-	-
All PPIs [8]							***11***	***11***	***1***	***3***
**ETFDH**	**Q16134**	(2gmh_A)	94	100/100/100	Homo 2-mer	-	-	-	-	-
ZNF581	Q9P0T4	(1ubd_C)	53	100/100	Monomer	-	-	-	-	-
MYH7B	A7E2Y1	(4p7h_B)	40	0/100/100	Monomer	-	-	-	-	-
KRTAP13-2	Q52LG2	-	-	-	-	-	-	-	-	-
OTX1	P32242	(2dms_A)	21	100/0	Monomer	-	-	-	-	-
KRTAR11-1	Q8IUC1	-	-	-	-	-	-	-	-	-
HSD17B10	Q99714	1u7t_B	98	100/100/100	Homo 4-mer	-	-	-	-	-
AIRE	O43918	1xwh_A<br>2lri_C	12	100/12/0	Monomer	-	-	-	-	-
TRIM69	Q86WT6	(2iwg_B)	36	0/0/100/100/0/<br>0	Monomer	-	-	-	-	-
**FAH**	**P16930**	(1hyo_A)	99	100/100	Homo 2-mer	-	-	-	-	-
EGFR	P00533	3qwq_A<br>3w32_A	51	100/100/100/<br>100/100/100	Monomer<br>Homo 2-mer	-	-	-	-	-
TCF4	P15884	2kwf_B	3	0	Monomer	-	-	-	-	-
KRTAP5-9	P26371	-	-	-	-	-	-	-	-	-
KRTAP10-8	P60410	-	-	-	-	-	-	-	-	-
ADAMTSL4	Q6UY14	-	-	-	-	-	-	-	-	-
SERTAD1	Q9UHV2	-	-	-	-	-	-	-	-	-
**FCGR2A**	**P12318**	1h9v_A	54	100/100/100/<br>100	Homo 2-mer<br>Monomer	1fcg	0	0	1	0
UBQLN1	Q9UMX0	(1j8c_A)<br>2jy5_A	18	100/100/98	Monomer	-	-	-	-	-
APCS	P02743	4avv_A	92	100	Homo 5-mer	3d5o	0	0	0	0
IGHG1	P01857	1hzh_H	100	100/100/100/<br>100	Homo 2-mer	3ry6	0	0	0	1

CRP	P02741	3l2y_A	92	100/100	Homo 5-mer	-	-	-	-	-
All PPIs [8]							***0***	***0***	***1***	***0***
**GALT**	**P07902**	5in3_B	87	91/99	Homo 2-mer	5in3	1	2	18	9
GRPEL1	Q9HAV7	(4ani_A)	76	98	Homo 4-mer	-	-	-	-	-
TNK2	Q07912	4hzs_D 1cf4_B	31	95/62/95/100/ 0	Monomer	-	-	-	-	-
TRIP13	Q15645	5vqa_A	85	88/84/86	Monomer	-	-	-	-	-
NQO1	P15559	1gg5_A	100	100	Homo 2-mer	-	-	-	-	-
KRTAP6-2	Q3LI66	-	-	-	-	-	-	-	-	-
All PPIs [8]							***0***	***2***	***17***	***9***
**GCDH**	**Q92947**	1siq_A	89	100/100/100	Homo 4-mer	1siq	9	11	19	7
NOS3	P29474	(1tll_A) 5uoc_A	55	100/100/99 /100	Homo 2-mer	-	-	-	-	-
RNF32	Q9H0A6	(1iym_A)	12	100/96/100/ 100/96/100/ 100/0/100/0	Monomer	-	-	-	-	-
APOE	P02649	2l7b_A	95	100	Monomer	-	-	-	-	-
CDC37	Q16543	5fwp_E 2n5x_A	68	99/88/100	Monomer	-	-	-	-	-
PSEN1	P49768	5fn5_B	48	54	Monomer Homo 2-mer	-	-	-	-	-
GRB2	P62993	1gri_A	97	88/100/97/ 100/100/100	Homo 2-mer	-	-	-	-	-
A2M	P01023	4acq_C	95	96/100/100/ 100/100/100	Homo 4-mer	-	-	-	-	-
EXOC6	Q8TAG9	(2a2f_X)	40	90	Monomer	-	-	-	-	-
ECSIT	Q9BQ95	-	-	-	-	-	-	-	-	-
All PPIs [8]							***9***	***9***	***18***	***4***
**HADHA**	**P40939**	(2wtb_A)	94	100/100/100/100	Monomer	N/A				
IKBKE	Q14164	(4im0_A)	90	100/100	Homo 2-mer	-	-	-	-	-
TRAF6	Q9Y4K3	3hcs_A	30	12/100/100/ 100/100/100 /100/	Homo 2-mer	-	-	-	-	-
PRKAB1	Q9Y478	5ezv_D	69	100/100	Hetero 3-mer	-	-	-	-	-
GABARAP	O95166	1kot_A	100	100/100	Monomer	-	-	-	-	-
BCAR3	O75815	3t6a_C	37	0/100	Monomer	-	-	-	-	-
GABARAPL1	Q9H0R8	5lxi_B	100	100/100	Homo 2-mer	-	-	-	-	-
HSD17B10	Q99714	1u7t_B	98	100/100/100	Homo 4-mer	-	-	-	-	-
EPB41	P11171	1gg3_C	32	100/100/0/96/ 0/0	Monomer	-	-	-	-	-
HLA-B	P30480	4u1m_A	76	0/100/100	Monomer	-	-	-	-	-
MAP1LC3B	Q9GZQ8	2zjd_C	98	100/100	Monomer	-	-	-	-	-
GABARAPL2	P60520	4co7_B	100	100/100	Monomer	-	-	-	-	-
USP49	Q70CQ1	-	-	-	-	-	-	-	-	-
**HADHB**	**P55084**	(2iik_A)	89	100/100/100	Homo 2-mer	-	-	-	-	-
HSPB2	Q16082	(3j07_A)	94	100	Homo 24-mer	-	-	-	-	-
SMN1	Q16637	-	-	-	-	-	-	-	-	-
ANXA7	P20073	(1dm5_A)	64	100/100/100	Homo 6-mer	-	-	-	-	-
TNNI3K	Q59H18	(1n11_A) 4yfi_C	42	93/100/100/0/ 100/100/100/ 100/100/100/ 100/100/100/ 100/100	Monomer Homo 2-mer	-	-	-	-	-
DUX1	O43812	(1fjl_A)	36	0/100	Homo 2-mer	-	-	-	-	-
TK1	P04183	1w4r_A	74	100	Homo 4-mer	-	-	-	-	-
RCC1	P18754	1a12_C	95	100/100/100/1 00/100/100/10 0/100	Homo 3-mer	-	-	-	-	-
CDKN1A	P38936	4rjf_B	12	0	Hetero 6-mer	-	-	-	-	-
GABARAPL1	Q9H0R8	5lxi_B	100	100/100	Homo 2-mer	-	-	-	-	-
HSD17B10	Q99714	1u7t_B	98	100/100/100	Homo 4-mer	-	-	-	-	-
MAP1LC3C	Q9BXW4	2ncn_A	86	100	Monomer	-	-	-	-	-

GRB7	Q14451	(3hk0_A)	47	100/100	Homo 2-mer	-	-	-	-	-
CDKN1A	P38936	-	-	-	-	-	-	-	-	-
**HBB**	**P68871**	1dxt_D	100	100	Homo 2-mer	1bbb	0	1	1	3
HP	P00738	(1gpz_A)	91	100	Monomer	4wjg	1	2	6	6
HBZ	P02008	3w4u_C	99	100	Homo 2-mer	3w4u	2	2	15	9
HBA1	P69905	1bz1_C	100	100	Homo 2-mer	2w72	2	3	17	12
SELENOT	P62341	-	-	-	-	-	-	-	-	-
All PPIs [8]							***1***	***3***	***17***	***8***
**HCFC1**	**P51610**	4go6_D	8	0	Homo 4-mer	4go6	5	4	1	0
GABPB1	Q06547	(1n11_A)	85	100/100/100/<br>100/100/100/<br>100/100/100	Monomer	-	-	-	-	-
CLP1	Q92989	(4ohx_A)	97	100/100/100	Monomer	-	-	-	-	-
MAGI1	Q96QZ7	2kpl_A<br>2q9v_A<br>2r4h_C<br>2yse_A<br>2kpl_A<br>2bpu_A<br>(1ex7_A)	9	100/97/100/0/<br>100/3/7/100/<br>100/100	Monomer	-	-	-	-	-
ASF1B	Q9NVP2	(2hue_A)	80	99	Monomer	-	-	-	-	-
E2F1	Q01094	2aze_B	23	100	Monomer	-	-	-	-	-
E2F3	O00716	(1cf7_A)	14	97/0	Monomer	-	-	-	-	-
E2F4	Q16254	5tuu_B<br>1cf7_A	26	98/100	Monomer	-	-	-	-	-
OGT	O15294	4xif_B<br>1w3b_A	67	100/100/100/<br>100/100/100/<br>100/100/100/<br>100/100/100/<br>100/100/100/<br>100/100/100/<br>100/100/100/<br>100/78/100/<br>100/100/100/<br>100/100/100	Monomer<br>Homo 2mer	-	-	-	-	-
FOXO3	O43524	2k86_A<br>2lqi_B	15	46/30/100	Monomer	-	-	-	-	-
THAP1	Q9NVV9	2jtg_A	41	100	Monomer	-	-	-	-	-
SIRT1	Q96EB6	5btr_A	49	100	Monomer	-	-	-	-	-
SIN3A	Q96ST3	2n2h_B<br>2l9s_B<br>2rms_B	10	0/46/100/100/<br>0	Monomer	-	-	-	-	-
FHL3	Q13643	2ehe_A<br>2cuq_A	29	100/24/100/7	Monomer	-	-	-	-	-
THAP11	Q96EK4	2lau_A	26	100	Monomer	-	-	-	-	-
ZBTB17	Q13105	4u2m_D<br>2lvr_A<br>2n26_A<br>2n25_A<br>2m0f_A<br>2m0d_A<br>2lvu_A	30	100/52/56/56/<br>52/54/4/100/<br>100	Monomer	-	-	-	-	-
FHL2	Q14192	2d8z_A<br>2miu_A<br>1x4l_A<br>1x4k_A	25	100/100/100/<br>100	Monomer	-	-	-	-	-
RNF2	Q99496	4r8p_L<br>3ixs_A	76	100/100/100/<br>100/100	Monomer<br>Homo 2-mer	-	-	-	-	-
TET2	Q6N021	5d9y_A	21	39	Monomer	-	-	-	-	-
NFE2L1	Q14494	-	-	-	-	-	-	-	-	-
SP1	P08047	-	-	-	-	-	-	-	-	-
PDCD2	Q16342	-	-	-	-	-	-	-	-	-
USP53	Q70EK8	-	-	-	-	-	-	-	-	-
BAP1	Q92560	-	-	-	-	-	-	-	-	-
THAP7	Q9BT49	-	-	-	-	-	-	-	-	-

CREBZF	Q9NS37	-	-	-	-	-	-	-	-	-
CREB3	O43889	-	-	-	-	-	-	-	-	-
TET3	O43151	-	-	-	-	-	-	-	-	-
All PPIs [8]							***5***	***2***	***1***	***0***
**HMGCL**	**P35914**	2cw6_A	91	100	Homo 2-mer	2cw6	0	1	0	0
RNF126	Q9BV68	(1x4j_A)<br>2n9p_A	21	100/100/100/<br>100/100/100/<br>100/100/100	Monomer	-	-	-	-	-
ADAMTS10	Q9H324	(1lsl_A)	10	0	Monomer	-	-	-	-	-
DNAJA1	P31689	2lo1_A<br>(1nlt_A)	58	100/100/100	Monomer	-	-	-	-	-
HSD17B10	Q99714	1u7t_A	98	100/100/100	Homo 4-mer	-	-	-	-	-
HES1	Q14469	2mh3_A	25	0/100	Homo 2-mer	-	-	-	-	-
GTF2B	Q00403	5iyb_M	98	100/100/100/<br>100	Monomer<br>Hetero 21-mer	-	-	-	-	-
ARL6IP1	Q15041	-	-	-	-	-	-	-	-	-
MS4A7	Q9GZW8	-	-	-	-	-	-	-	-	-
All PPIs [8]							***0***	***1***	***0***	***0***
**HPD**	**P32754**	3isq_A	96	100/100/100	Homo 2-mer	3isq	1	5	1	0
CDKN1A	P38936	4rjf_B	12	0	Monomer<br>Hetero 6-mer	-	-	-	-	-
IKBKG	Q9Y6K9	4bwn_A<br>3brt_D	20	98/92	Homo 2-mer	-	-	-	-	-
All PPIs							***1***	***5***	***1***	***0***
**HSD3B2**	**P26439**	(3wj7_A)	95	100	Homo 3-mer	-	-	-	-	-
**IVD**	**P26440**	1ivh_A	91	100/100/100/<br>100	Homo 4-mer	1ivh	1	2	5	3
ACTN3	Q08043	(4d1e_A)	95	100/100/100/<br>100/100/100/<br>100/100	Homo 2-mer	-	-	-	-	-
GPSM3	Q9Y4H4	-	-	-	-	-	-	-	-	-
All PPIs [8]							***1***	***2***	***3***	***2***
**LMBRD1**	**Q9NUN5**	-	-	-	-	-	-	-	-	-
**MCCC1**	**Q96RQ3**	(3u9s_A)	92	100/100/100/100	Hetero 12-mer	N/A				
**MCCC2**	**Q9HCC0**	(3u9r_B)	95	100	Homo 6-mer	-	-	-	-	-
HSPB2	Q16082	(3j07_A)	94	100	Homo 24-mer	-	-	-	-	-
**MLYCD**	**O95822**	4f0x_A	92	100/100	Homo 2-mer<br>Homo 4-mer	2ygw,<br>4f0x	1	3	2	4
UBALD1	Q8TB05	(2dzl_A)	33	100	Monomer	-	-	-	-	-
RBM5	P52756	2lkz_A	12	0/0/100/0	Monomer	-	-	-	-	-
All PPIs [8]							***1***	***2***	***2***	***2***
**MMAA**	**Q8IVH4**	2www_D	75	89	Homo 2-mer	2www	1	2	0	2
All PPIs [8]							***1***	***2***	***0***	***2***
**MMAB**	**Q96EY8**	2idx_C	72	98	Homo 3-mer	2idx	0	1	3	1
SPERT	Q8NA61	-	-	-	-	-	-	-	-	-
All PPIs [8]							***0***	***1***	***2***	***1***
**MMACHC**	**Q9Y4U1**	3sc0_A	84	100	Monomer	N/A				
MTR	Q99707	4ccz_A<br>(3bul_A)	48	100/100/100/<br>92/100	Monomer	-	-	-	-	-
MMADHC	Q9H3L0	5cv0_A	57	62	Homo 2-mer	-	-	-	-	-
CREB5	Q02930	-	-	-	-	-	-	-	-	-
**MMADHC**	**Q9H3L0**	5cv0_A	57	62	Homo 2-mer	5cv0	3	8	0	0
MMACHC	Q9Y4U1	3sc0_A	84	100	Monomer	-	-	-	-	-
All PPIs [8]							***3***	***8***	***0***	***0***
**MTHFR**	**P42898**	(1v93_A)	45	100	Monomer	N/A				
LSM8	O95777	(4c92_A)	96	100	Monomer<br>Hetero 7-mer	-	-	-	-	-

**MUT**	**P22033**	3bic_	95	99	Homo 2-mer	2xiq	6	8	11	3
PCBD1	P61457	1dcp_A	95	100	Homo 4-mer	-	-	-	-	-
All PPIs [8]							***6***	***8***	***10***	***2***
**PAH**	**P00439**	(5den_A)	95	100	Homo 2-mer	1j8u, 5fii	0	3	13	12
QDPR	P09417	1hdr_A	97	100	Monomer	-	-	-	-	-
PCBD1	P61457	1dcp_A	95	100	Homo 4-mer	-	-	-	-	-
All PPIs [8]							***0***	***2***	***13***	***11***
**PAX8**	**Q06710**	2k27_A	35	100/0	Monomer	N/A				
UNK	Q9C0B0	5elh_A<br>(5elk_A)	17	100/100	Homo 2-mer	-	-	-	-	-
ANXA7	P20073	(1dm5_A)	64	100/100/100/<br>100	Homo 6-mer	-	-	-	-	-
CXCL9	Q07325	(1qnk_A)	56	100	Homo 2-mer	-	-	-	-	-
GCM2	O75603	(1odh_A)	31	100	Monomer	-	-	-	-	-
HOXC8	P31273	(2r5y_A)	31	100	Monomer	-	-	-	-	-
AES	Q08117	(4om3_A)	59	87	Homo 4-mer	-	-	-	-	-
HOXC9	P31274	2msy_A	26	0/100	Monomer	-	-	-	-	-
SAE1	Q9UBE0	1y8r_A	91	93	Monomer,<br>Hetero 3-mer	-	-	-	-	-
SMAD4	Q13485	1dd1_B<br>3qsv_A	45	100/96	Homo 2-mer,<br>Homo 3-mer	-	-	-	-	-
HGS	O14964	4avx_A<br>3f1i_H	28	100/100/100	Monomer	-	-	-	-	-
CHUK	O15111	5ebz_A	88	100/100/0	Homo 6-mer	-	-	-	-	-
NCL	P19338	2krr_A<br>2fc9_A<br>2fc8_A	24	100/100/100	Monomer	-	-	-	-	-
PKM	P14618	3gr4_A	98	100/100	Homo 4-mer	-	-	-	-	-
EP300	Q09472	4bhw_A<br>1l3e_B<br>3io2_A<br>(1tot_A)	23	82/100/100/0/<br>0/100/100/100	Homo 2-mer	-	-	-	-	-
POU2AF1	Q16633	1cqt_J	9	9.2	Monomer	-	-	-	-	-
RB1	P06400	4elj_A<br>2aze_C	64	100/98/28/93	Monomer	-	-	-	-	-
RPLP2	P05387	4beh_B	100	100	Monomer,<br>Hetero 2-mer	-	-	-	-	-
CDK3	Q00526	-	-	-	-	-	-	-	-	-
CLSTN1	O94985	-	-	-	-	-	-	-	-	-
SS18L1	O75177	-	-	-	-	-	-	-	-	-
LONRF1	Q17RB8	-	-	-	-	-	-	-	-	-
RSBN1	Q5VWQ0	-	-	-	-	-	-	-	-	-
TMA16	Q96EY4	-	-	-	-	-	-	-	-	-
SERINC1	Q9NRX5	-	-	-	-	-	-	-	-	-
**PCCA**	**P05165**	(3n6r_A)	91	100/100/100/<br>100/100/100/<br>100	Monomer,<br>Hetero 12-mer	N/A				
ERRFI1	Q9UJM3	4zjv_C	10	47	Monomer	-	-	-	-	-
PCCB	P05166	(3n6r_B)	93	100	Homo 6-mer	-	-	-	-	-
PARP2	Q9UGN5	-	-	-	-	-	-	-	-	-
MCC	P23508	-	-	-	-	-	-	-	-	-
**PCCB**	**P05166**	(3n6r_B)	93	100	Homo 6-mer	-	-	-	-	-
PCCA	P05165	(3n6r_A)	91	100/100/100/<br>100/100/100/<br>100	Monomer,<br>Hetero 12-mer	-	-	-	-	-
ACTN3	Q08043	(4d1e_A)	95	100/100/100/<br>100/100/100/<br>100/100	Homo 2-mer	-	-	-	-	-
CSNK2B	P67870	4dgl_B	93	100	Monomer	-	-	-	-	-
**SLC22A5**	**O76082**	-	-	-	-	-	-	-	-	-
SLC9A3R2	Q15599	-	-	-	-	-	-	-	-	-
PDZD3	Q86UT5	-	-	-	-	-	-	-	-	-
SLC9A3R1	O14745	-	-	-	-	-	-	-	-	-

MTUS2	Q5JR59	-	-	-	-	-	-	-	-	-
NOTCH2NL	Q7Z3S9	-	-	-	-	-	-	-	-	-
KRT27	Q7Z3Y8	-	-	-	-	-	-	-	-	-
**SLC25A13**	**Q9UJS0**	4p5w_A	46	0/0/0	Homo 2-mer	4p5w	1	9	4	3
USP50	Q70EL3	(2hd5_A)	88	100/100	Monomer	-	-	-	-	-
All PPIs [8]							***1***	***9***	***4***	***3***
**SLC25A20**	**O43772**	(1okc_A)	95	100/100/94	Monomer	N/A				
**TAT**	**P17735**	3dyd_A	85	0/100	Homo 2-mer	3dyd	2	1	0	1
UBE3A	Q05086	1c4z_A<br>2kr1_A	40	100/98	Monomer	-	-	-	-	-
GLUL	P15104	2ojw_C	97	100/100	Homo 10-mer	-	-	-	-	-
JPT2	Q9H910	(4hpo_L)	97	100	Monomer	-	-	-	-	-
All PPIs [8]							***2***	***1***	***0***	***1***
**TGFB1**	**P01137**	5ffo_H	83	95/94	Monomer,<br>Homo 2-mer	5ffo	1	3	3	1
CDKN2A	Q8N726	(2g7l_A)	98	100	Homo 2-mer	-	-	-	-	-
KLK9	Q9UKQ9	(2psx_A)	91	100	Monomer	-	-	-	-	-
FSTL1	Q12841	(1lr7_A)	22	100/100/100	Monomer	-	-	-	-	-
CCL5	P13501	(1el0_A)	80	100	Monomer	-	-	-	-	-
BMPR1A	P36894	(3mdy_A)<br>2k3g_A	62	100/100/100/<br>100	Monomer	-	-	-	-	-
MMP9	P14780	(1ck7_A)	96	100/100/100/<br>100/100/100/<br>100/100	Monomer	-	-	-	-	-
LCE3B	Q5TA77	(3tjq_A)	93	99	Monomer	-	-	-	-	-
BUB1	O43683	(3e7e_A)<br>2lah_A	32	100/100/100/<br>100	Monomer	-	-	-	-	-
DIRAS3	O95661	(2p67_A)	95	100/100/100	Monomer	-	-	-	-	-
AXIN2	Q9Y2T1	(1dk8_A)<br>(1wsp_A)	17	0/100/100/11	Homo 2-mer<br>Homo 3-mer	-	-	-	-	-
TGFBR3	Q03167	(3qw9_A)	20	51	Homo 2-mer	-	-	-	-	-
THBS1	P07996	(1yo8_A)<br>5foe_B<br>2es3_A<br>3r6b_A	53	100/100/98/10<br>0/100/100/100<br>/100/100/100/<br>100/96	Monomer	-	-	-	-	-
THRSP	Q92748	(3ont_A)	94	95	Homo 2-mer	-	-	-	-	-
MEOX2	P50222	(1puf_A)	19	100	Monomer	-	-	-	-	-
LAMB1	P07942	(4aqs_A)	26	100/100/72/0/<br>0/0/0/0/0/0/0/<br>0/100	Monomer	-	-	-	-	-
PRSS50	Q9UI38	(1z8g_A)	86	100/100	Monomer	-	-	-	-	-
HOXC6	P09630	(1ftz_A)	28	100	Monomer	-	-	-	-	-
MTA3	Q9BTC8	(4bkx_A)	28	0/0/0/100	Monomer	-	-	-	-	-
HOXA1	P49639	(1b72_A)	27	100	Monomer	-	-	-	-	-
LCE3D	Q9BYE3	(1m8n_A)	99	100	Monomer	-	-	-	-	-
NSD3	Q9BZ95	(4yz8_A)<br>4rxj_A	15	0/100/100	Homo 2-mer	-	-	-	-	-
CST6	Q15828	(2ch9_A)	85	100	Homo 2-mer	-	-	-	-	-
ERBB2	P04626	3n85_A<br>3pp0_B	48	99/100/99/92/<br>100/97	Monomer	-	-	-	-	-
TGFBR1	P36897	3faa_E<br>2l5s_A	66	100/100/100/<br>100	Monomer	3kfd	0	0	0	0
KLK7	P49862	2qxj_A	88	100	Monomer	-	-	-	-	-
AGR3	Q8TD06	3ph9_B	81	100	Monomer	-	-	-	-	-
WFIKKN1	Q96NZ8	2ddi_A	13	0/0/0/0/0/<br>100/0/0/0	Monomer	-	-	-	-	-
LAMTOR5	O43504	3msh_A	99	100	Homo 2-mer	-	-	-	-	-
ITGAV	P06756	3ije_A	89	93/100/100	Monomer,<br>Hetero 2-mer	5ffo	1	3	0	0
FNTA	P49354	2h6i_A	83	100/100/100/<br>100/100	Monomer,<br>Hetero 2-mer	-	-	-	-	-
LPXN	O60711	1x3h_A<br>(1v6g_A)	21	5/100/100/100	Monomer	-	-	-	-	-

		1m3v_A)								
TLR2	O60603	2z7x_A 1fyx_A	70	98/98/100/100 /100/100/100	Monomer, Hetero 2-mer	-	-	-	-	-
APC	P25054	3nmz_A 1emu_B 2rqu_B 1deb_B 1m5i_A	14	0/0/0/0/0/0/0/ 0/0/100/0/100/ 0/0/0/50/96/93	Monomer, Hetero 4-mer	-	-	-	-	-
PDLIM2	Q96JY6	3pdv_A	25	100/0/0	Monomer	-	-	-	-	-
DAXX	Q9UER7	2kzs_A	13	98	Monomer	-	-	-	-	-
SNAI1	O95863	3w5k_B	42	92/100	Monomer, Hetero 2-mer	-	-	-	-	-
FBXW7	Q969H0	2ovq_B	63	100/100/100/ 100/100/100	Monomer, Hetero 2-mer	-	-	-	-	-
DLC1	Q96QB1	3kuq_A 2gky_A	13	100/100/0	Monomer	-	-	-	-	-
FKBP1A	P62942	3mdy_B	100	100	Monomer, Hetero 2-mer	-	-	-	-	-
AURKA	O14965	2j4z_B	67	100/100	Monomer	-	-	-	-	-
ST14	Q9Y5Y6	4jyt_A	28	100/0/0/0/0/0/ 0/0	Monomer	-	-	-	-	-
EGFR	P00533	3qwq_A 3w32_A	51	100/100/100/ 100/100/100	Monomer	-	-	-	-	-
TGFBR2	P37173	5e8y_A 1plo_A	53	99/99/100	Monomer	3kfd	0	0	0	0
BCL10	O95999	2mb9_A	45	100	Monomer	-	-	-	-	-
APP	P05067	3nyl_A 3ktm_C 1aap_A 2roz_A 2lp1_A	25	79/55/97/100/ 100/100	Monomer	-	-	-	-	-
ENG	P17813	5hzv_A	86	99	Monomer	-	-	-	-	-
MMP2	P08253	1gxd_A	94	100/100/100/ 100/100/100/ 100/100	Monomer, Hetero 2-mer	-	-	-	-	-
ITGB6	P18564	5ffo_B	87	100/100/100/0	Monomer	5ffo	0	1	0	0
LCE1C	Q5T751	-	-	-	-	-	-	-	-	-
WFIKKN2	Q8TEU8	-	-	-	-	-	-	-	-	-
ANGPTL4	Q9BY76	-	-	-	-	-	-	-	-	-
KLK5	Q9Y337	-	-	-	-	-	-	-	-	-
CYSRT1	A8MQ03	-	-	-	-	-	-	-	-	-
FCHO1	O14526	-	-	-	-	-	-	-	-	-
MCC	P23508	-	-	-	-	-	-	-	-	-
KRTAP10-8	P60410	-	-	-	-	-	-	-	-	-
KRTAP1-1	Q07627	-	-	-	-	-	-	-	-	-
LRRC32	Q14392	-	-	-	-	-	-	-	-	-
DIP2A	Q14689	-	-	-	-	-	-	-	-	-
PDGFRL	Q15198	-	-	-	-	-	-	-	-	-
GREB1	Q4ZG55	-	-	-	-	-	-	-	-	-
LCE1D	Q5T752	-	-	-	-	-	-	-	-	-
LCE1A	Q5T7P2	-	-	-	-	-	-	-	-	-
LCE3A	Q5TA76	-	-	-	-	-	-	-	-	-
LCE2C	Q5TA81	-	-	-	-	-	-	-	-	-
ADAMTSL4	Q6UY14	-	-	-	-	-	-	-	-	-
BLID	Q8IZY5	-	-	-	-	-	-	-	-	-
CATSPER1	Q8NEC5	-	-	-	-	-	-	-	-	-
SCGB3A1	Q96QR1	-	-	-	-	-	-	-	-	-
KRTAP4-11	Q9BYQ6	-	-	-	-	-	-	-	-	-
BCAS3	Q9H6U6	-	-	-	-	-	-	-	-	-
PSMC3IP	Q9P2W1	-	-	-	-	-	-	-	-	-
MLH3	Q9UHC1	-	-	-	-	-	-	-	-	-
LCE2B	O14633	-	-	-	-	-	-	-	-	-
All PPIs							***2***	***7***	***3***	***1***
**TSHR**	**P16473**	2xwt_C	31	0/100/100	Monomer	N/A				

SCRIB	Q14160	4wyt_A 1x5q_A 1wha_A	12	100/100/100 /100/0/0/0/ 100	Monomer	-	-	-	-	-
RABAC1	Q9UI14	-	-	-	-	-	-	-	-	-

[1] Gene name and UniProt code for the proteins associated to diseases detected in newborn screeening (first line, in bold) and for their interacting partners as provided by Interactome3D database (rest of lines).

[2] PDB codes and chain IDs corresponding to the available 3D structure of the individual proteins analyzed here and to their interacting partners (in parenthesis, the template of the homology model provided by Interactome3D, if no experimental structure is available).

[3] Global structural coverage: percentage of the sequence of each individual protein that has available 3D structure in the corresponding PDB files.

[4] Domain structural coverage: structural coverage (%) of each of the individual domains annotated for each individual protein.

[5] Oligomeric state for each individual protein as indicated in the biological unit in the PDB.

[6] PDB code of the structure of the complex formed by the individual proteins analyzed here with each interacting partner, as provided by Interactome3D. The first line corresponding to each individual protein analyzed here refers to the self-interactions (generated by the oligomeric state indicated in the biological unit).

[7] Mutated residues (involved in neutral or pathogenic mutations) that are located in each interface region as indicated by the complex structure. In the case of homo oligomers, a mutated residue could be counted in different regions if so appears in the different subunits.

[8] Global summary of the location of mutations in all the interactions for a given protein. Each mutated residue is counted only at one region.
